# Machine learning of language use on Twitter reveals weak and non-specific predictions

**DOI:** 10.1038/s41746-022-00576-y

**Published:** 2022-03-25

**Authors:** Sean W. Kelley, Caoimhe Ní Mhaonaigh, Louise Burke, Robert Whelan, Claire M. Gillan

**Affiliations:** 1 School of Psychology, Trinity College Dublin, Dublin, Ireland; 2 Trinity College Institute of Neuroscience, Trinity College Dublin, Dublin, Ireland; 3 Global Brain Health Institute, Trinity College Dublin, Dublin, Ireland

**Keywords:** Human behaviour, Human behaviour

## Abstract

Depressed individuals use language differently than healthy controls and it has been proposed that social media posts can be used to identify depression. Much of the evidence behind this claim relies on indirect measures of mental health and few studies have tested if these language features are specific to depression versus other aspects of mental health. We analysed the Tweets of 1006 participants who completed questionnaires assessing symptoms of depression and 8 other mental health conditions. Daily Tweets were subjected to textual analysis and the resulting linguistic features were used to train an Elastic Net model on depression severity, using nested cross-validation. We then tested performance in a held-out test set (30%), comparing predictions of depression versus 8 other aspects of mental health. The depression trained model had modest out-of-sample predictive performance, explaining 2.5% of variance in depression symptoms (*R*^2^ = 0.025, *r* = 0.16). The performance of this model was as-good or superior when used to identify other aspects of mental health: schizotypy, social anxiety, eating disorders, generalised anxiety, above chance for obsessive-compulsive disorder, apathy, but not significant for alcohol abuse or impulsivity. Machine learning analysis of social media data, when trained on well-validated clinical instruments, could not make meaningful individualised predictions regarding users’ mental health. Furthermore, language use associated with depression was non-specific, having similar performance in predicting other mental health problems.

## Introduction

Approximately 20% of adults will experience a mental illness in any given year^1^. But our ability to treat those affected is hampered by the fact that patients present to clinics relatively infrequently^2^, and when they do so, it is often belated, making their symptoms more difficult to treat^3^. For this reason, efforts to detect mental illness early and predict individual vulnerability is a key focus of research. Of course, this is challenging in real-world settings, because it is unclear what sources of data can and should be utilised to make these predictions. It has been proposed that one way to overcome this difficulty is to use other sources of data that the general public produce regularly, such as social media data, to detect, predict and better understand mental health in the population. Social media adoption is widespread with approximately 72% of US adults using at least 1 social media platform offering a unique opportunity for gathering information about mental health^4^.

Recent studies have suggested that social media data can be used to recognise a broad range of mental health problems in the general public including depression^5–10^, eating disorders^11–14^, schizophrenia^15–17^, and suicide^18–21^. A key premise of such work is that these data could be used to facilitate early intervention, for example by providing users with personalised risk scores for having a mental illness and/or developing one in the near future. Inherent in that is the assumption that such models are (i) accurate enough to be clinically actionable and (ii) precise enough to detect one illness from another. In the present paper, we investigated the extent to which models based on social media data meet these criteria.

There is now a wealth of data supporting the notion that people with depression use language differently than those without depression. For example, depressed individuals use more first person singular pronouns^22^, obscenities^6^ and express more negative emotions^5^ in their language. This language occurs in a variety of settings including semi-structured interviews^23,24^, journal entries^22,25^, and critically, social media posts^7,9,26^. However, it is not clear if these language patterns are specific to depression. Shared variance between disorders presents a challenge to identifying what aspects of language use are specific to a particular disorder. Indeed, because mental health disorders tend to co-occur in the same individuals and our existing diagnostic system lacks clear separation between disorders^27,28^, language-based models are unlikely to have high specificity when trained on summed scores or diagnostic categories. Studies typically compare data from depressed individuals to that of healthy controls, but do not test if language patterns discriminate among psychiatric disorders. There are few clear distinguishing features^29–32^ between mental health conditions in the few papers that have studied multiple groups. For example, although elevated first person singular pronoun usage is considered to be a defining feature of language in depression, – reflecting an increase in self-focused attention—it is also elevated in people with obsessive-compulsive disorder^30,31^, anxiety^30,31^, eating disorders^11–13,30,33^, and schizophrenia^15,16,30,34,35^. Without accounting for comorbidity among disorders, it is not possible to discern whether first-person singular pronouns are unique to depression, a transdiagnostic marker of mental illness, or better explained by another aspect of mental health entirely. Tackling the issue of specificity more directly, one study found greater evidence of third-person plural pronouns (they, them) in those who participated in Schizophrenia discussion forums versus other sorts of mental health forums, a putative marker of persecutory delusions^31^. However, the topic of the discussion forum from which language use was gathered is a major confounding factor. That is, the content in these forums may not reflect speech patterns of persons with schizophrenia in their everyday life, when not discussing their illness. Moreover, no clinically validated screening tools were used to define cases, rather, participation in these forums and explicit statements of self-diagnosis were used to identify patients.

As discussed in a recent review^36^, this is a common approach and has been applied to study language-use on more generic social media outlets like Twitter, using ‘statements of diagnosis’ e.g., “I have PTSD”^29,32^, to define cases of mental illness, rather than validated clinical instruments. In addition to the issue of diagnostic validity, this approach is limited by the fact that a person who openly reveals a diagnosis on Twitter is not someone trying to conceal it and is probably not part of the cohort of undiagnosed/untreated individuals that such methods may wish to identify. Moreover, they may be more likely to tweet about disorder-relevant topics, which could create circularity, inflating effect sizes, leading us to conclude that mental health status is more readily detected from social media data than it actually is. For example, Coppersmith et al. reported 85% precision at detecting generalised anxiety disorder on Twitter when allowing for false-positive rate of 10%^29^ using this method. To remove these potential circularities, studies are moving toward less biased methods, where mental health status is not defined by the same or similar content that is ultimately used to study language use. For example, separating the content used to define disorder status (e.g. membership of a mental health forum) from content used to characterise language use (posts by those users on other forums)^37^. When this approach was taken, accuracy was substantially worse. Using a range of machine learning algorithms, the F1 score (average of precision and recall) rarely exceeded 0.5^30^. This poor performance could be because the signal is weak, or the diagnoses are not accurate. However, even in studies with a more rigorous definition of disorder status, because the cases are still binary, evaluating how specific language use is to that disorder and not another condition remains an issue. This is due to the lack of multiple continuous measures of mental health in the sample participants. In the few studies that have administered self-report questionnaires to consenting participants, performance was again modest, albeit somewhat improved^9^, but the specificity of the findings to the disorder of study (here, depression) was not examined.

Thus, while social media makes substantial amounts of language data available to researchers, a caveat to much of this research is the acquisition of high-quality mental health data. The notion that we can detect mental illness from social media posts presents opportunities for public health interventions, but with this comes significant privacy concerns and potential for discriminatory practices to emerge^38^. But are these opportunities and concerns overstated? To date there is little evidence that mental health status can be detected accurately, and even less evidence for individuals who do not choose to openly disclose/discuss their mental health disorder status online. Moreover, if such predictions could be made with any fidelity, it is unclear if they can be in any way specific, which is crucial if these indicators are to be used to guide the choice of intervention. The present study sought to address these issues, determining (i) the specificity of language patterns to different aspects of mental health and (ii) providing an estimate of the performance of these models on unseen data. To do this, we acquired Twitter data over the past year from over 1000 individuals who completed 9 different self-report mental health-related questionnaires and consented for us to link that to their Tweets. We tested the performance of a machine learning algorithm trained on a gold-standard ground-truth measure of depression symptomatology when applied to unseen data. To test its specificity, we then applied this depression model to predicting scores of a range of mental health phenotypes. Finally, we trained a machine learning model to predict the residuals of three transdiagnostic dimensions of mental health (after controlling for one-another), allowing us to identify text features that are specific to a particular dimension after removing the shared variance between disorders.

## Results

Age was significantly negatively associated with all psychiatric questionnaires, except alcohol abuse (all *β* < 0.07, *p* < 0.05). Female participants had significantly elevated eating disorder symptoms (*β* = 0.34, SE = 0.07, *p* < 0.001), social anxiety (*β* = 0.38, SE = 0.07, *p* < 0.001), generalised anxiety (*β* = 0.28, SE = 0.07, *p* < 0.001), and depression (*β* = 0.35, SE = 0.07, *p* < 0.001) than men. Male participants had a significantly higher rate of alcohol abuse symptoms (*β* = 0.31, SE = 0.01, *p* < 0.001) (Supplementary Fig. 1). As expected, all psychiatric questionnaires were positively correlated with each other (Supplementary Fig. 2).

### Univariate associations with mental health symptoms

**Fig. 1:**
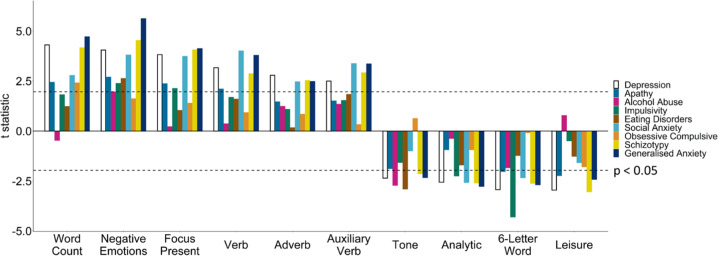
Associations between 9 self-reported psychiatric questionnaires and top 10 text features associated with depression. Associations between nine self-reported psychiatric questionnaires and mean values over the past year of the top ten LIWC text features associated with depression severity, controlling for age and gender (*n* = 1006). Dashed lines indicate *p* values below 0.05.

The top ten Linguistic Inquiry and Word Count (LIWC) text features associated with depression were word count, negative emotions, focus on present, verbs, adverbs, auxiliary verbs (all positively associated with depression severity, *β* > 0.08, *p* < 0.05) and tone, the analytic summary variable, number of six-letter words, and leisure words (all negatively associated with depression severity, *β* < −0.07, *p* < 0.05). These effects were non-specific. Negative emotions (all *β* > 0.08, *p* < 0.001) were significantly positively associated with all aspects of mental health studied, except alcohol abuse (*β* = 0.05, SE = 0.03, *p* = 0.05) and obsessive-compulsive disorder (*β* = 0.04, SE = 0.03, *p* = 0.11). Schizotypy, social anxiety, and generalised anxiety were significantly associated with all 10 text features (all *β* > |0.06|, *p* < 0.05), except for the associations between social anxiety with tone (*β* = −0.03, SE = 0.03, *p* = 0.33) and leisure (*β* = −0.04, SE = 0.03, *p* = 0.12), which were non-significant. None of the alternative questionnaires were significantly associated with a text feature in the opposite direction of depression. Individual text features were thus not specific to depression but broadly associated with other psychiatric dimensions (Fig. 1).

**Fig. 2:**
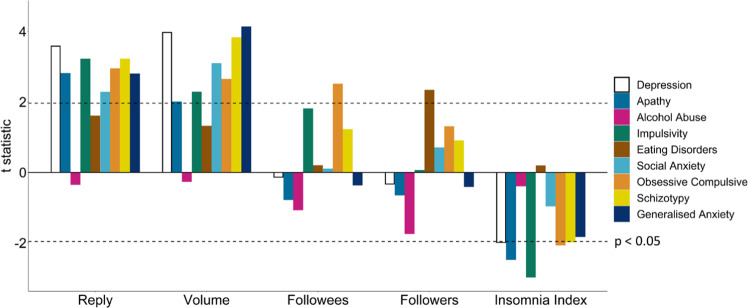
Associations between 9 self-reported psychiatric questionnaires and Twitter metadata features. Replies (all *β* > 0.08, *p* < 0.05) and volume (all *β* > 0.03, *p* < 0.05) of tweets were significantly elevated across all aspects of mental health studied, except for alcohol abuse and eating disorders (*n* = 1,006). Participants with elevated obsessive-compulsive symptomology tended to follow more accounts (*β* = 0.03, SE = 0.01, *p* = 0.01). While participants with more eating disorder symptoms had significantly more followers (*β* = 0.02, SE = 0.01, *p* = 0.02). Participants with greater depression, apathy, impulsivity, obsessive-compulsive, and schizotypy symptoms tweeted more at night than during the day (all *β* < −0.06, p < 0.05). Dashed lines indicate *p* values below 0.05.

In terms of Twitter metadata, participants with elevated obsessive-compulsive symptoms followed more accounts (*β* = 0.03, SE = 0.01, *p* = 0.01), while participants who scored higher on eating disorder severity had a larger number of followers (*β* = 0.02, SE = 0.01, *p* = 0.02). Participants scoring high on depression, apathy, impulsivity, obsessive-compulsive disorder, and schizotypy tended to tweet more at night, i.e. higher insomnia index (all *β* < −0.06, *p* < 0.05). Replies to Tweets (all *β* > 0.08, *p* < 0.05) and volume of Tweets (all *β* > 0.03, *p* < 0.05) were positively associated with all aspects of mental health recorded, except alcohol abuse and eating disorders (Fig. 2).

### Machine learning

We trained an Elastic Net model on depression symptoms and tested it on unseen data. The model of depression symptomatology had an *R*^2^ of 0.025 (*r* = 0.16) vs. *R*^2^ −0.040 (*r* = −0.16) for the null model. An extended model trained on LIWC text features plus age and gender (*R*^2^ = 0.045, *r* = 0.22) performed better than a model with randomised text features plus age and gender (*R*^2^ = 0.039, *r* = 0.20). Our simulation results also demonstrated that we were sufficiently powered to detect a larger signal, if it was truly present (see Supplementary Material).

**Fig. 3:**
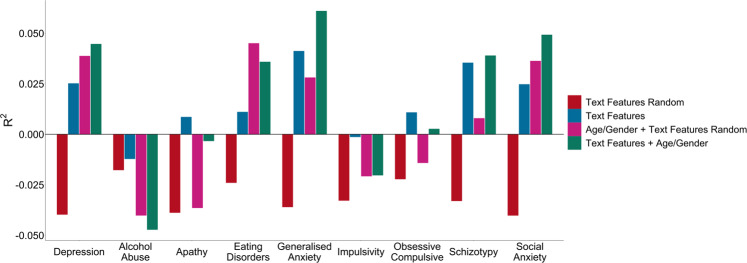
Elastic Net predictive performance of a depression model tested on itself, and 9 other aspects of mental health. Predictive performance (*R*^2^) from an Elastic Net model trained on depression and tested on each of the other aspects of mental health recorded for randomised text features (red), text features only (blue), age and gender plus randomised text features (pink), and text features plus age and gender (green) (*n* = 1006).

After establishing there was modest, but non-zero signal, we applied the depression trained model using LIWC text features only to the other eight psychiatric scales to test for specificity (Fig. 3). The depression model had above zero predictive power for all other aspects of mental health except impulsivity and alcohol abuse. Nominally, the depression model performed somewhat worse when tested on apathy (*R*^2^ = 0.008, *r* = 0.11), alcohol abuse (*R*^2^ = −0.012, *r* = 0.04), eating disorder symptoms (*R*^2^ = 0.011, *r* = 0.12), and obsessive-compulsive disorder symptoms (R^2^ = 0.011, *r* = 0.12), predictive ability was identical to depression for social anxiety (*R*^2^ = 0.025, *r* = 0.16) and the model performed nominally better in predicting schizotypy (*R*^2^ = 0.035, *r* = 0.19) and generalised anxiety (*R*^2^ = 0.041, *r* = 0.21) scores. Alcohol abuse and impulsivity were the only aspects of mental health that had negative *R*^2^ values for the non-random models. Increasing the number of words per user, while maintaining a constant sample size, did increase the depression trained model’s predictive performance. Increasing the threshold from 5 days of Tweets (minimum of 43 words) to 500 words per user caused the *R*^2^ to increase from 0.010 to 0.034 (Supplementary Table 1). However, there was substantial variation at the lower word count thresholds with *R*^2^ = −0.001 at 200 words per user up to a maximum of *R*^2^ = 0.044 at 400 words per user.

Prior research has suggested that partially dissociable transdiagnostic dimensions of mental health may be a better fit to the underlying neurobiology of mental illness. This was not true of its fit to language patterns in Twitter data assessed here. A model trained to predict ‘anxious-depression’ scores performed nominally worse (*R*^2^ = 0.016) than the models trained and tested on the depression (*R*^2^ = 0.025) or generalised anxiety (R^2^ = 0.045) questionnaires, as reported above (Supplementary Fig. 3). The anxious-depression model was also non-specific, having modest but non-zero predictive power for both ‘compulsivity and intrusive thought’ (*R*^2^ = 0.025) and social withdrawal (*R*^2^ = 0.014). To test if there were any systematic characteristics of those subjects who were poorly predicted by the model, we examined the residuals of our main depression model. There were no significant associations between any aspect of Twitter use, e.g., number of Tweets, and depression residuals from the held-out test set (all |*β*| > 0.02, *p* > 0.05) (Supplementary Fig. 4a). Additionally, depression model residuals were normally distributed and centred on zero (Mean = −0.05, *t* = −0.84 (df = 301), *p* = 0.40) (Supplementary Fig. 4b). Deviations from true depression scores, i.e., depression residuals, within the LIWC text features model are not due to any systematic differences in participant engagement on Twitter.

**Fig. 4:**
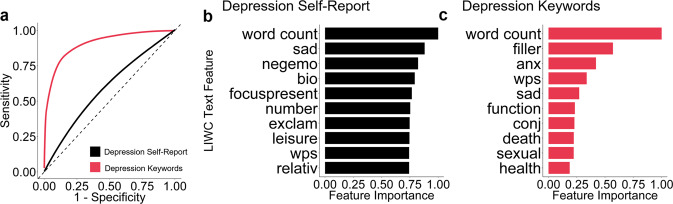
Comparison of text feature only random forest models trained on a definition of depression derived from (i) a depression self-report questionnaire and (ii) a person’s use of depression-relevant keywords in their Tweets. **a** Receiver operator curves for depression self-report (AUC = 0.56) and depression keyword (AUC = 0.83) trained models, dashed line indicates chance level performance (*n* = 1006). **b** Top ten text features for the depression self-report model. **c** Top ten text features for the depression keyword model.

To test if our results were specific to our choice of machine learning method, we also evaluated the predictive performance of a case-control depression-trained classification model using a previously established cut-off for depression (Table 1). The best performing model was a support vector machine (SVM) trained on data from the top 476 users by word count which had an AUC and accuracy of 0.59. There was no difference in performance between the SVM and random forest (RF) models, and both models performed worse than the 68% accuracy found in prior work^5^. Decreasing the sample size to include only the top 476 participants also had no effect on predictive performance, although there was a substantial increase in sensitivity with a slight decline in specificity. Finally, we examined how classification performance changes when we define depression case-ness from Twitter data itself, using keywords. The depression-keyword model had an 83.6% accuracy, 0.83 AUC, 76.9% sensitivity, and 88.9% specificity, which substantially outperformed the classification model trained and tested on self-reported depression (57% accuracy, 0.57 AUC, 52% sensitivity, and 63% specificity) (Fig. 4). While participants with depression-relevant keywords do have greater depression severity (*β* = 0.26, SE = 0.016, *z* = 4.00 (df = 1004), *p* < 0.001), the use of keywords within posts to define cases of mental illness demonstrates a substantial overestimation of model performance compared to validated self-report questionnaires.

**Table 1:** Depression classification performance of a support vector machine (SVM) and random forest (RF) model with varying sample size.

Model	AUC	Accuracy	F1	Sensitivity	Specificity
SVM (*n* = 1,006)	0.56	0.59	0.42	0.33	0.79
SVM (n = 476)	0.59	0.59	0.54	0.52	0.66
RF (*n* = 1,006)	0.56	0.58	0.44	0.38	0.75
RF (*n* = 476)	0.57	0.57	0.53	0.52	0.63
De Choudhury SVM (*n* = 476)	NA	0.68	NA	0.58	NA

Depression classification performance was similar between SVM and RF regardless of sample size. In the reduced sample size (*n* = 476) of participants with high word counts there was an increase in the F1 score and sensitivity for both the SVM and RF models. However, in both models the increase in sensitivity was accompanied by a reduction in specificity. Neither model improved over the classification performance of de Choudhury et al.^5^.

### Comparison of models trained on Tweets, Retweets, Likes

By training the depression model on Tweets, Retweets, and Likes separately, we found that the Likes model had the greatest predictive value (*R*^2^ = 0.026) compared with an *R*^2^ of 0.010 for the Tweets only model (Supplementary Fig. 5a). The improved predictive power may be because there was simply more data for Likes (*M* = 768.4, SD = 1041.3) compared to Tweets (*M* = 231.3, SD = 489.4) and Retweets (*M* = 173.5, SD = 416) (Table 2). Indeed, when we split data based on quantity, predictive power was greatest in the 4th quartile of Tweet data (*R*^2^ = 0.043) with negative *R*^2^ values from models trained on the 1st and 2nd quartiles of Tweets (Supplementary Fig. 5b). Therefore, improved performance can be achieved when the corpus is particularly large, though it clearly remains modest, explaining just 4% of variance in depression.

**Table 2:** Twitter use and demographics of sample.

Twitter behaviour	Mean (SD)
No. of Tweets	231.3 (489.4)
No. of Retweets	173.5 (416)
No. of Likes	768.4 (1041.3)
Word count per day	96.5 (119.9)
Age	Mean (SD)
Years	30.5 (10.1)
Gender	*N* (%)
Male	312 (31%)
Female	668 (66.4%)
Transgender Male	6 (0.6%)
Transgender Female	1 (0.1%)
Non-Binary	16 (1.6%)
Other	3 (0.3%)
Country	*N* (%)
Ireland	32 (3.2%)
United Kingdom	412 (41%)
United States	472 (46.9%)
Canada	52 (5.2%)
Australia	24 (2.4%)
Other	14 (1.4%)
Education	*N* (%)
Less than high school	22 (2.2%)
High School	220 (21.9%)
Some University	301 (29.9%)
Bachelor’s degree	325 (32.3%)
Master’s degree	108 (10.7%)
Professional degree	17 (1.7%)
Doctorate	13 (1.3%)
Employment status	*N* (%)
Currently employed	642 (63.8%)

### Similarity and specificity of text features across mental health phenotypes

**Fig. 5:**
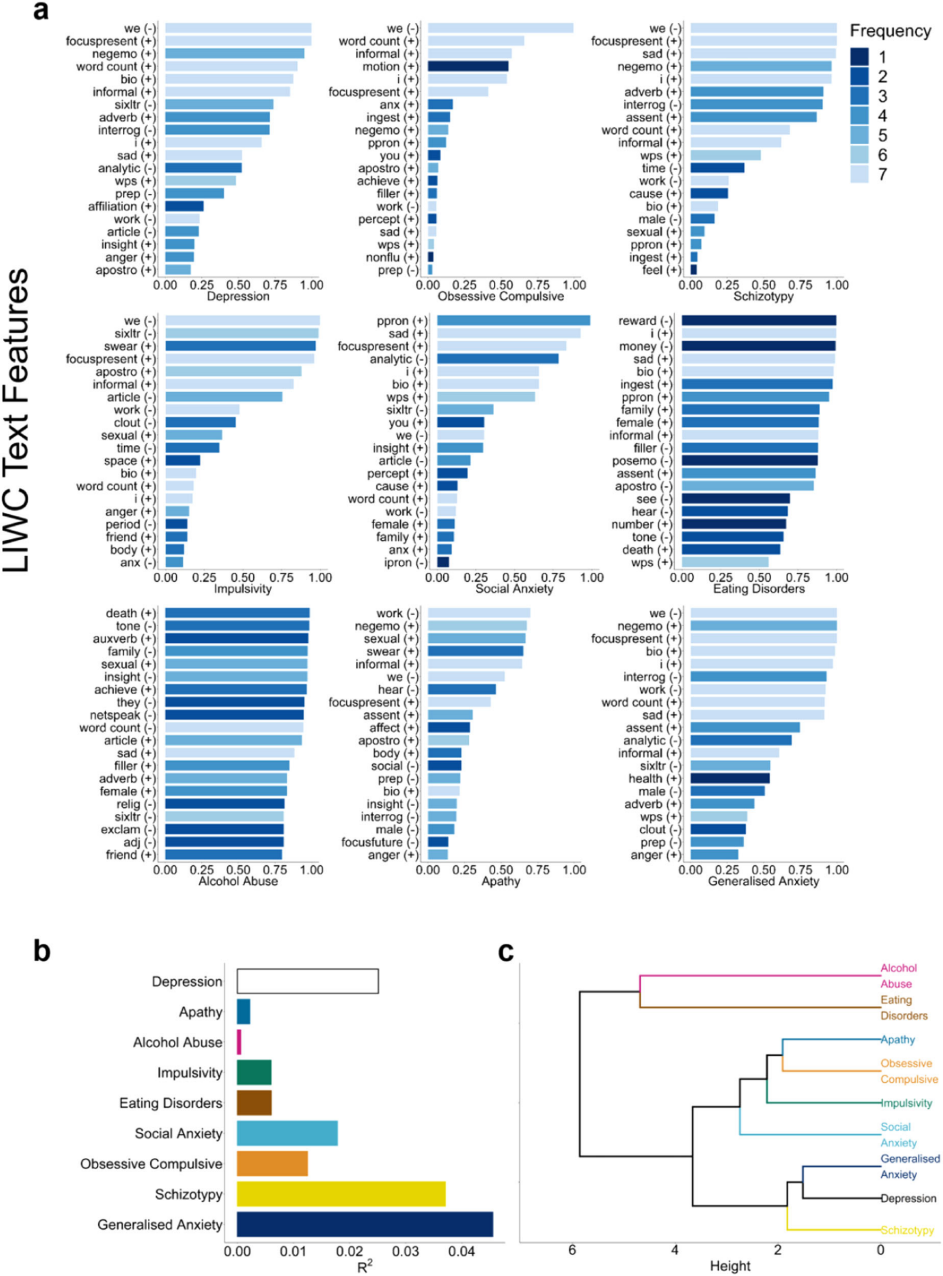
Depression model text feature selection frequencies and dendrogram of language similarities between aspects of mental health. **a** Model selection frequencies for the top 20 text features in models of 9 psychiatric questionnaires over 100 iterations. Darker colours indicate text features that appear among the top 20 text features across fewer questionnaires, i.e., are more specific. The mean direction of association between each text feature and the target outcome is denoted by a + (positive) or – (negative) next to the label. **b** Predictive performance of models trained and tested on each self-report questionnaire. **c** Hierarchical clustering dendrogram from a model trained on mean text feature selection frequencies.

To compare the content of models developed for each of our nine aspects of mental health, we generated predictive models for each disorder separately and examined how similar the most predictive LIWC text features were. In the depression-trained model, we found that ‘focus on present’ and 1st person plural pronouns were selected in 100% of models. While negative emotions and 1st person singular pronouns, text features previously found to be associated with depression, had selection frequencies of 0.951 and 0.659 respectively (Fig. 5a). First-person singular pronouns were selected more often in models related to schizotypy (0.963), social anxiety (0.658), eating disorders (0.999) and generalised anxiety (1.0) than depression. Negative emotions were slightly more specific to depression than 1st person pronouns with higher selection frequencies in only schizotypy (0.963) and generalised anxiety (1.0). Among the top 20 text features by selection frequency, only affiliation words were unique to depression. Eating disorders and alcohol abuse had the largest number of unique text features with five and six respectively. The generalised anxiety trained model had the highest predictive performance of all models (*R*^2^ = 0.045), followed by schizotypy (*R*^2^ = 0.037). For impulsivity, eating disorders, alcohol abuse, and apathy the percent of variance explained was below 1% (Fig. 5b).

Hierarchical clustering revealed, unsurprisingly given the high correlation across these questionnaires, that depression language use was most similar to generalised anxiety followed closely by schizotypy (Fig. 5c). Obsessive-compulsive disorder was most closely related to apathy in terms of language use patterns and are slightly more dissimilar to each other compared to the depression, generalised anxiety, and schizotypy cluster. Perhaps most interestingly, alcohol abuse and eating disorders formed their own cluster largely separate from other disorders, indicating substantial differences in language use both between those disorders and relative to the other disorders considered.

**Fig. 6:**
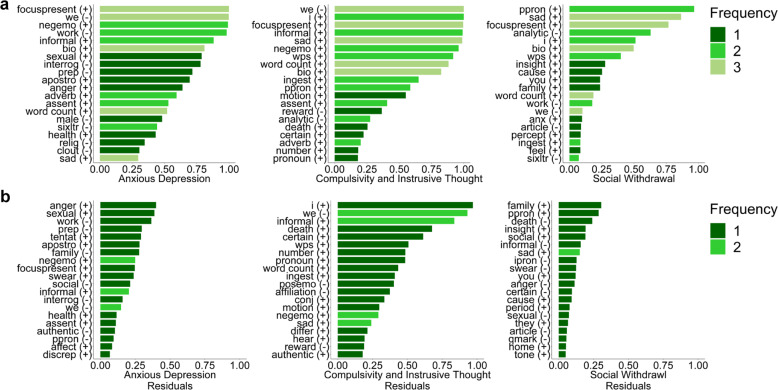
Transdiagnostic dimension text feature selection frequencies. **a** Model selection frequencies for the top 20 text features in models of 3 transdiagnostic dimensions over 100 iterations. **b** Model selection frequencies for the top 20 text features in models trained on the residuals of each transdiagnostic dimension after controlling for the shared variance due to the other dimensions.

To examine specificity, we focused on a smaller set of three transdiagnostic dimensions of mental health that can be derived from the larger set of questionnaires: ‘anxious-depression’, ‘compulsivity and intrusive thought’ and ‘social withdrawal’. Much like the analysis of the original questionnaire total scores, the top text features by selection frequency in each transdiagnostic dimension were not specific to any one dimension (Fig. 6a). Similar to models trained on each questionnaire individually, 1st person plural pronouns and focus on present words were among the top text features associated with not just anxious-depression, but also and compulsivity and intrusive thought dimensions. In each dimension, less than 45% of the top 20 text features were specific to the top 20 for that particular dimension. However, after removing the shared variance between dimensions, at least 80% of text features were specific to each dimension (Fig. 6b). No text feature was present in the top 20 of all 3 dimensions. Anger words, 1^st^ person singular pronouns, and family relevant words were the top text features associated with anxious-depression, compulsivity and intrusive thought, and social withdrawal respectively. Importantly each of these text features was specific to that particular dimension, i.e., not found in the top 20 for any other dimension.

## Discussion

There is growing interest in the power of artificial intelligence for improving healthcare provision, early intervention, and diagnosis. But large amounts of data are needed to develop and train models, which can be arduous to gather. Social media data has been suggested to be a convenient and readily available source of such data. This is because social media platforms are in widespread use, users produce high volumes of data, regularly and spanning many years, and these data often contain rich personal and emotional information of putative relevance to their mental state. Although several studies have examined this in recent years, there are substantial limitations to the methods in widespread use^36,39^, including but not limited to the validity of diagnostic classifications employed and the rigour of the machine learning methods employed. Here, we collected Twitter data and 9 validated self-report questionnaires from over 1000 participants assessing their mental health. We used gold-standard machine learning methods with out-of-sample testing to establish the predictive power of models trained to predict depression and other aspects of mental health, using linguistic features derived from Tweets.

A model developed to predict individual differences in self-report depression explained 2.5% of variance when tested out of sample. The age and gender model, however, slightly outperformed the text feature-only model, illustrating that a similar level of depression prediction can be achieved using just these two data points. It is worth noting that age and gender are not routinely available on Twitter but were gathered as part of our survey. When age, gender, and text features were included in the same model, it still only explained approximately 4% of variance in depression severity. We examined the specificity of this depression model, on 8 other questionnaire total scores gathered from the same participants. We found that although the model had some small predictive value for 6 other aspects of mental health studied here, generalised anxiety, schizotypy, obsessive-compulsive disorder, eating disorder, apathy and social anxiety, it was not able to explain variance in alcohol abuse and impulsivity. Furthermore, we found that there were no associations between any aspect of Twitter use, e.g., word count, and the residuals of the depression model’s predictions. Failures in model performance, therefore, seem to be random and not explained by lower engagement nor number of social connections.

We tested if previously identified transdiagnostic symptom dimensions, which tend to perform better than these questionnaires in fitting cognitive test performance^40^, might improve signal and/or specificity. This was not the case. After controlling for shared variance among the transdiagnostic dimensions, we found that most text features were specific to the residuals of each dimension. Perhaps most strikingly, 1st person singular pronouns have been consistently found to be a key characteristic of depression-relevant language^22,41^, but when controlling for shared variance, we found that an increased use of 1st person pronouns was actually most associated with the compulsivity and intrusive thought dimension. Overall, generalised anxiety and schizotypy were the best performing models while the alcohol abuse model had close to zero out-of-sample performance. Hierarchical clustering revealed that language use associated with alcohol abuse and eating disorders were most dissimilar to the other disorders. Most prior social media research has focused on associations between language use and alcohol usage at the group, rather than individual, level^42^. A possible explanation for the low predictive value of the alcohol model is that few people in our study scored high enough to qualify as alcohol dependent.

A depression classification model trained on the presence of depression-relevant keywords had substantially better predictive performance compared to a model trained on dichotomised self-reported depression on a validated instrument. Prior studies have shown that regular expression, i.e., keywords, can be used to identify depression with a high degree of accuracy^43,44^. To our knowledge, however, no studies have compared relative predictive performance of a depression-keyword-trained model to one trained on depression self-report scores within the same sample. Although self-report measures are more difficult to pragmatically acquire from a large sample, they represent an important and clinically validated ground truth. Our results indicate the potential pitfalls of defining cases of mental illness through keyword-based methods, that is, a sort of content-based circularity can arise when social media posts are used to define caseness, train and evaluate machine learning models. Our data suggest that persons more likely to discuss depression in Tweets, have a distinct pattern of associated language use, but they do not necessarily suffer from clinical depression, with only 50% of these participants meeting the clinical cut-off for depression. These findings underscore the need to use valid ground truth estimates of mental health in developing models of clinical relevance.

Exploratory analyses found that elevated rates of replying and Tweeting were broadly associated with mental health, correlating with all questionnaire total scores, except alcohol abuse and eating disorders. Inconsistent evidence exists around whether people with greater depression severity are more^8^ or less^5^ active on social media. We found that participants with greater obsessive-compulsive severity had more followees while people with more severe eating disorder symptoms had more account followers. Depressed individuals have consistently been shown to Tweet more at night than during the day^5,45,46^. Later Tweet times were associated with depression severity, but also apathy, impulsivity, obsessive compulsive symptoms, and schizotypy. Impulsivity had the strongest association with the insomnia index, in line with prior research showing a positive association between impulsivity and sleep disturbances^47,48^. Besides findings related to the number of followees and followers, Twitter metadata, like language use, was generally not specific to any one mental health condition.

People with depression have been found to use language differently from healthy controls. Most studies, however, compare people with one mental health disorder to healthy controls^22,25,49–51^; few have examined the specificity of different aspects of language use across disorders. The non-specific patterns of language use observed here, both in prior work^29–31^ and the current study, is likely related to the high comorbidity rates among disorders. We found that only by removing the shared variance among disorders could we identify which aspects of language use were specific to each mental health dimension. Major depressive disorder is positively associated with a variety of other mental health conditions including panic disorder, agoraphobia, generalised anxiety disorder, post-traumatic stress disorder, obsessive-compulsive disorder, and separation anxiety disorder^52^. For example, a patient diagnosed with major depression is 8.2 times more likely to have a concurrent diagnosis of generalised anxiety than someone without depression^53^. In our study, we found that depression and anxiety had the most similar language use of any pair of disorders. Depression symptoms overlap strongly with other disorders and are associated with numerous symptoms in other diagnostic categories^54^. In a network of Diagnostic and Statistical Manual of Mental Disorders-IV symptoms, depression symptoms (insomnia, psychomotor agitation/retardation, and depressed mood) were the most connected symptoms with connections to over 28% of other symptoms in the network^55^. The spread of symptoms across disorders makes it unlikely that individual text features or even combinations of text features could ever be specific to categorical disorders, a finding in line with the growing consensus that these diagnostic categories are overlapping and warrant revision^28^.

Social media is not a one-way street. While the content of social media posts reflects the underlying mental health of the user, interactions, both passive and active, on the platforms can act to either improve or worsen mental health. When users experience a stressful event, they are more likely to disclose this information on social media. Self-disclosure was shown to subsequently moderate the adverse effects of a stressful event and led to enhanced life satisfaction and lower depression via enhanced social support^56^. However, in a separate study, Reddit users who transitioned to talking about suicide had elevated levels of self-disclosure but received less social support and engagement than users who did not^57^. Furthermore, specific types of social support are more likely to lead to improvements in mental health, e.g., use of the phrase ‘be tough’^58^. Increasing awareness about these types of comments would help friends, family, and content moderators to know what to say to and what not to say to someone experiencing mental health difficulties. While there are benefits to self-disclosure these can only be realised if the user is able to communicate free of stigma and receive adequate support. The effects of self-disclosure on social media highlight the need to follow users longitudinally and consider factors beyond just language use, i.e., social network structure, when predicting mental health. Considering the availability of online social support could help triage users with the same predicted risk of mental illness; users with less social support should be prioritised for receiving help.

Most Twitter data are generated by a small subset of users, 80% of Tweets are written by only 10% of users^59^. We found some evidence that machine learning language models perform slightly better when trained on subsets of users with more Tweets. This might suggest that in an even more select sample, e.g., those in the top 10% of users overall, one could produce more reliable predictions. However, two things are important to remember here. First, even in our top quartile, the variance explained only rose to a high of 4.3%, additional gains are unlikely to take this to the realm of real-world clinical utility. Similarly increasing the minimum word count per user only slightly increased the percent variance explained. At a minimum threshold of 400 words, 6.4% of variance was explained, while a threshold of 500 words was slightly worse at 3.4%. Second, those users are not representative of social media users in general, so even if such performance could be achieved, these models are unlikely to be generalisable. An interesting possibility is that the signal may be more meaningfully improved if private sources of text could be harnessed such as text messages. This would have the additional benefit of increasing the amount of data available for each user while simultaneously being more relevant to a user’s true mental health status.

Although we demonstrated that social media data has low predictive power at an individual level, this should be contextualised as part of the broader landscape of effect sizes in mental health science. For example, well-established correlates of mental health problems such as adverse childhood experiences only yield an area under the curve of 0.58 in predicting mental health problems at age 18^60^. A recent preprint showed that resting-state and structural brain-wide associations to psychopathology are exceedingly small, with no reliable correlation exceeding 0.16^61^. Because these observations do not have value as individual predictors, does not make the observation devoid of meaning. Mental health is exceedingly complex and likely combinations of a range of sources of multimodal data will be required to take these small effects and transform them into meaningful N-of-1 predictions. Twitter data, by itself, has already proven an interesting testbed for nascent theories of mental health such as network theory, which for example, has struggled to acquire large enough longitudinal datasets to test some of its core predictions^62^. We recently found for example that using social media posts as a proxy for experience sampling allowed us to study a large cohort of individuals through a transition to a depressed state, detecting subtle network signatures of depression vulnerability^63^.

Mental health detection from social media offers the potential for generating continuous insights into mental health at the population and individual level, but also poses a unique set of ethical challenges. Large-scale analyses of social media data are typically exempt from requiring participant consent due to the public nature of data and lack of experimental intervention. Because of this exemption, social media users are often unaware of whether or not their data are included in research and when asked, tend to be uncomfortable with the idea that their Twitter data could be used for research purposes without their knowledge^64^. While it is impractical to ask for consent in all circumstances, requiring consent whenever possible ensures that participants have safeguards for how their data is used. Predicting an individual’s mental health outside of a clinical context inherently poses the question of how to act on that information and whether there is in fact an obligation to act^65^. Unlike clinicians, software developers are not obligated to intervene if their algorithm detects that a person is struggling with their mental health. If the developers are not obligated to intervene, would the burden fall on family members, friends or the individuals themselves? Even if a patient consented to having their social media feed monitored by their physician, a high rate of false positives could overwhelm a clinician and impede their ability to effectively allocate care. Furthermore, there is a potential for misuse of mental health predictions by bad actors who do not consider the best interests of the user. Passive and automatic detection of mental illness could lead to targeted advertisements of prescription medication^66^ or result in an increase in health insurance premiums. A final concern relates to algorithmic bias based on the data used to train these models. Social media users tend to be younger, more affluent, and hold more left leaning political views than the general population^59,67^. Furthermore, social media research is strongly focused on predominantly English-speaking countries yet there is evidence that people from different cultures behave differently online, for example, users from China and India post questions online more frequently than users from the US and UK^68^. Extrapolating models to very different users than those the models were trained on could lead to systematic biases that impact the predictive performance for groups not included in the training data.

Prior studies have had larger Twitter datasets in terms of the number of posts per user. For example, de Choudhury et al.^5^ had a mean of 4500 posts per user in a 1-year period, while our study had a mean of about 1100 posts, including likes, per user. As mentioned above, models perform better when they are provided with more training data per user. Indeed, this study achieved greater predictive power than reported here. However, there were other differences across our studies too; our sample was twice as big, and we used an independent training set to build our model and then evaluated it on an independent test set. Compared to simple K-fold cross-validation using the entire dataset^5^, this procedure is less likely to overfit the data and overestimate predictive performance. Another potential limitation to our study is that our text features analysis was restricted to only using categories from the LIWC library. Some evidence exists that more data-driven approaches, e.g., topic analysis, could slightly improve predictive ability over closed libraries^18,69^. More sophisticated machine learning models, such as convolutional neural networks have the potential to make superior predictions than more commonly used algorithms, although with the limitation of needing substantially more data^70^. While these methods might indeed yield improvements in performance, the use of LIWC has key advantages. LIWC is a closed library that has been well-validated and studied across a range of communication media from diary entries^22^ to spoken word^71^. This means that the numerical values and classifications assigned to individual words in LIWC does not change from dataset to dataset, as is often the case with topics and neural networks^72,73^. This makes the insights derived here more reproducible and generalisable to new datasets that may be of keen interest in the future, such as text messages and email communications.

Regarding the choice of social media platform, it is nonetheless a limitation that our study was confined to Twitter. Recent evidence has also shown that Facebook may be more predictive of mental health conditions than Twitter^74^. We selected Twitter because it is the most used social media platform for studying mental health, comprising approximately 40% of studies on the subject, while Facebook makes up only about 8%^36^. It remains a limitation that these results could reflect a relative lack of predictive performance that is particular to Twitter. Because we did not have binary diagnostic information, we did not attempt to classify participants with either depression vs. anxiety, obsessive-compulsive disorder etc., i.e., multi-class classification of mental health diagnoses. Instead, we tried to continuously predict a participant’s score on a range of self-report questionnaires probing different aspects of mental health. Therefore, rather than differentiating users with one diagnosis or another, we instead attempted to quantify the similarity of language use between self-report symptoms of highly comorbid conditions. We think this dimensional approach has many advantages, but this creates a limitation in how directly these data can be applied to diagnoses assigned by a clinician. Finally, subjects in this study reported mental health symptoms at the point of study entry, and we analysed data corresponding to the 12-month period directly prior to this. This necessitates taking a ‘trait’ perspective on the mental health symptoms we assessed and it is likely that our model is diluted by variations in state/episodic features of depression. However, in a recent study, we found that individuals’ use of depression-relevant text features in fact didn’t change significantly across within-subject periods of mental health and wellness, suggesting this may not be a major issue^63^.

We found that language use patterns on Twitter that relate to depression symptom severity cannot be used to develop predictive models with high accuracy on an individual subject basis. A model trained to predict depression is also non-specific, being additionally predictive of several mental health symptom profiles. Although performance was poor at the individual subject level, the effect sizes observed are not out of proportion with other routinely studied cross-sectional observations in psychiatry. The addition of age and gender improved performance of our depression model, suggesting that the combination of various sources of multimodal data (with individually small effect sizes) is a viable path forward to improve predictive power of these class of models. Furthermore, controlling for other mental health conditions and training models on the resultant residuals is a promising method for finding aspects of language use specific to that condition. To our knowledge, we are the first study to train machine learning algorithms on the residuals of mental health dimensions in order to identify unique patterns of language. This approach highlights the benefits of using self-report questionnaires to measure mental health since it is not possible for studies with a binary classification of cases, i.e., healthy control vs. case, to account for shared variance between disorders. Although classification studies are able to identify cases of mental illness, they are unlikely to be able to determine specifically what aspects of language are different and unique to a particular condition. Determining specific changes in language patterns and use is crucial for the utility of using text data for diagnostic purposes, regardless of data source.

Nevertheless, we do not believe that social media should be used in a diagnostic setting both for privacy concerns on behalf of the user and the relatively low quality of prediction. Despite the low signal, by virtue of the availability of large amounts of data, the analysis of social media data remains a useful tool to test theories of mental health that are difficult to test using conventional means. Should people be concerned that their mental health status can be unintentionally revealed by the content of their Tweets? We think the data do not support this as a meaningful risk at present.

## Methods

### Participants

We recruited 1450 participants for this study. The majority of participants were recruited on Clickworker (*N* = 1395), an online worker platform, and were paid €2.5 for their participation. A smaller number participated voluntarily (i.e., without payment) and were recruited through general advertising on Twitter and in print media (*N* = 55). Participants were included for analysis if they were at least 18 years old and had a Twitter account with at least 5 days of tweets and if at least 50% of their tweets were in English. They were also required to pass an attention check, a combination of a Captcha and an item with an obvious correct response (“Please select ‘A little’ if you are paying attention”). Of the 1450 participants recruited, 99 were excluded due to failing the attention check and a further 345 participants were excluded for either not having at least 5 days of tweets or fewer than 50% of their tweets were in English. After excluding these participants, 1006 participants were brought forward for analysis. Participants had a mean age of 30.5 years (SD: 10.1, range: 18–68), a majority were female (66.4%), currently employed (63.8%), and resided in either the UK (41%) or USA (46.9%). Participants tweeted an average of 21,126 words (SD: 30,204), median of 6432 words, and a range of 43–163,700. In total, there were 21,252,845 words posted across the 1006 participants.

### Procedure

After providing informed consent, participants were asked to complete a self-report questionnaire and provide their Twitter handle which was used to collect the most recent (max. 3200) tweets and (max. 3200) likes from their account. Tweets were collected using a data collection app written in Python using the Twitter developer’s Application Programming Interface. Participants were asked to provide their age, gender, country of residence, current employment status, and highest educational attainment. Participants then completed nine different psychiatric questionnaires including the Zung depression scale^75^, Short Scales for Measuring Schziotypy^76^, Obsessive Compulsive Inventory Revised^77^, Eating Attitudes Test (EAT-26)^78^, Barratt Impulsiveness Scale (BIS-11)^79^, Alcohol Use Disorders Inventory Test^80^, Apathy Evaluation Scale^81^, Liebowitz Social Anxiety Scale^82^, State-Trait Anxiety Inventory^83^ (Supplementary Fig. 6). This study was approved by the Trinity College Dublin Department of Psychology Research Ethics Committee (Approval ID: SPREC112018-32).

### Pre-processing and text analysis

We restricted our analysis to tweets published in the 12 months prior to survey completion. Before text analysis, extraneous information was removed from tweets including: reply symbol (@), hashtag symbol (#), emojis, punctuation, links (URLs), and all other non-alphanumeric characters. Periods, exclamation points, and question marks were the only punctuation retained because they are necessary to calculate the number of words per sentence. Tweets were aggregated into daily bins and text analysis was then performed on all tweets published per day per user. Daily observations were chosen to increase the amount of text for reliable estimation of text features. Text analysis of daily Tweets was carried out using the LIWC 2015 dictionary^84^. The LIWC is a dictionary comprised of approximately 6400 words and word-stems with 90 different output variables including: linguistic characteristics (e.g., articles and pronouns), psychological constructs (e.g., sadness and positive emotions), and general text information (e.g., punctuation and word count).

In addition to text features, we carried out some additional analyses using Twitter metadata variables including number of followees and followers, replies per day, number of tweets per day, and the insomnia index. The insomnia index is the relative difference in percentage of tweets tweeted during the day (6:01 a.m. to 8:59 p.m.) versus the night (9 p.m. to 6 a.m.). Previous research has found that people with depression tend to tweet more at night^26,46^.

### Univariate associations with mental health symptoms

We focus on depression at the outset because it is the most commonly studied disorder in this field of research and therefore several benchmark studies exist. To examine the specificity of text features to depression, in the first instance, we report univariate associations between the total score for each psychiatric disorder and the top 10 text features associated with depression severity including word count, negative emotions, focus on present, verbs, auxiliary verbs, adverbs, tone, analytic, six-letter words, and leisure words. Each linear model contained just one text feature and controlled for the effects of both age and gender (e.g., depression ~ adverbs + age + gender), both of which showed associations with mental health symptoms consistent with prior work (Supplementary Fig. 1).

### Machine learning

Next, we trained a model to predict depression scores from LIWC text features using Elastic Net regularisation^85^. Elastic Net is a combination of L-1 and L-2 norm regularisation, preforming both feature selection and regularisation which results in a sparse solution when features are correlated with each other. We chose Elastic Net because the input text features are highly correlated and it has been shown to make accurate predictions with small effect sizes in samples larger than 400^86^. Another advantage of Elastic Net is that the output (i.e. regression coefficients) is easily interpretable. It is thus possible to directly compare the relative importance of input features and see how they contribute to predictive performance.

We tested the model’s performance in predicting out-of-sample depression scores, and to assess specificity, we also tested it on out-of-sample scores on eight other psychiatric scales (which we did not use in training). The data was split into training (70%) and test (30%) sets, stratified by gender to ensure equal proportions of gender categories between the two sets. Nested cross-validation was performed within the training data, using 10 outer loops stratified by gender and five inner loops, with optimisation of Elastic Net hyperparameters (alpha and the l1 ratio) within the inner loops. We repeated this process 100 times to select the best Elastic Net model to take forward to test on the 30% of data we held out. We first tested it on depression scores to determine predictive power and then on the eight other psychiatric scales. Random label permutation was used to determine the predictive value of these models and to ensure that the apparent predictive power of LIWC was not simply the result of confounded associations between age, gender, mental health, and language use. We preformed control analyses that included: LIWC text features plus age and gender as features and compared it to a model with just age and gender (and randomly permuted LIWC features). This allowed us to determine if there was a marginal benefit of text features above and beyond the predictive ability of basic demographics^87^. Finally, we sought to identify reasons why our model made poor predictions for so many individuals. To do this, we examined depression residuals (i.e. each persons’ discrepancy between their true and predicted depression scores) and tested if these related to how participants use and interact with Twitter. We associated depression residuals from the held-out test set of the LIWC text feature only model with the z-score of (i) mean word count, (ii) total number of Tweets, (iii) tweet volume, (iv) total number of replies, (v) number of followers, and (vi) number of followees.

Selecting a machine learning method based in its performance is a form of overfitting. For this reason, we chose to work with the Elastic Net a priori, a method well suited to continuous prediction problems. However, for the purposes of comparison to other studies, and to ensure our results are not specific to the Elastic Net, we repeated our analysis pipeline using two alternative classification models. Area under the curve (AUC) is the primary measure of predictive performance in most studies in the literature, so to enable comparisons between our results and those of previous studies, we binarised depression scores and classified participants as either depressed or non-depressed. Participants with depression scores above 50 were classed as depressed, while those with scores under 50 were non-depressed^75^. We restricted our analysis to depression because that is the primary disorder of interest in the current study and several questionnaires used do not have established clinical cutoffs. Consequently, it would not be possible to directly apply the depression trained model to the other conditions as we did for the Elastic Net model. We next ran four separate models based on varying combinations of sample size and model type to assess performance. We used two classification models: random forest (RF) and support vector machine (SVM) with a radial kernel. Both models were validated with tenfold cross-validation and 100 experimental runs using either (i) the full sample of participants (*n* = 1006) or (ii) the top 476 participants by word count. We chose the second sample size to be the same as that from de Choudhury et al.^5^, so we could make a direct comparison between our classification performances. Our sample had fewer mean posts over the previous year compared to that of de Choudhury et al.^5^, a mean of 4533.4 posts per user compared to 1173.2 posts per user. By selecting the top 476 users by word count the number of posts per user increased to 2277.1, thereby making the samples more comparable.

### Comparison of validated self-report vs twitter-derived ground truth

We compared the performance of a model trained on self-reported depression versus depression keywords extracted from Twitter. Using a regular expression, i.e., depress*, we identified any posts that contained depression-relevant keywords and phrases. We found that approximately 2.0% of all days with Tweets had at least 1 keyword matching the depression regular expression. We then classified participants as either depressed or not depressed based on whether they had at least 1 post with a depression keyword present; with this approach, 44% of participants were classified as depressed. Days with Tweets that contained the depression keyword were omitted to ensure some independence between the testing and training data. Subsequently, we trained a RF classification model on the depression keyword outcome and compared it to the model trained on binarized self-reported depression. Finally, we evaluated all our models’ performance using AUC, F1 score, accuracy, sensitivity, and specificity.

### Transdiagnostic psychiatric dimensions

To test if performance might be more specific when using transdiagnostic psychiatric dimensions, rather than these questionnaire total scores, the individual answers to the 209 questions in our survey were transformed into 3 transdiagnostic dimensions. These were dimensions previously identified using factor analysis of these 9 questionnaires^88^, corresponding to ‘anxious depression’, ‘compulsivity and intrusive thoughts’, and ‘social withdrawal’. We then used the weights derived from that independent study to construct the 3 transdiagnostic dimensions in this study. The transdiagnostic dimensions are designed to reduce collinearity across these questionnaires and have been shown to relate to cognitive test performance and brain signatures in a stronger and more specific manner than the original questionnaire total scores^40,88,89^.

### Comparison of Tweets, Retweets, Likes

We also ran additional analyses to understand the influence of Tweet type i.e., Tweet, Retweet, Like, and the amount of Twitter data on model performance. We trained depression models using text features that included only Tweets, Retweets, or Likes and then tested the models on all nine questionnaire total scores. By training models separately on each type of Twitter data, we could determine whether each Tweet type is independently predictive of depression. We subsequently split the text feature data which contained Tweets, Retweets, and Likes merged together, into quartiles based on the total number of Tweets. Then, we trained a depression model on data from each quartile and determined its predictive performance. Splitting the data into quartiles also allowed us to control for sample size, such that any differences in performance are solely caused by the amount of text data. We expected that models trained on data from the upper quartiles (i.e., with the most Twitter data) would have better performance than the lower quartiles.

### Similarity and specificity of text features across mental health phenotypes

To probe specificity in more detail and with less of a central focus on depression, we generated predictive models for each psychiatric questionnaire separately, using the procedure outlined above. The Elastic Net can assign a weight of 0 to variables that are not predictive. To better understand our results, we used feature selection derived from the Elastic Net regularisation by summing each of the text feature’s non-zero weights within each main fold and then averaged this value over the 100 iterations. The ‘selection frequency’ provides a useful heuristic for the importance of each text feature as a predictor of that clinical phenotype. Selection frequency is a good measure of a variable’s importance since text features that are frequently included are more likely to be truly associated with the target outcome. After generating text feature selection frequencies for each questionnaire, we applied a hierarchical clustering algorithm, using Ward’s method, to determine how similar language use is between mental health conditions^90^. To examine the potential for specificity in a highly correlated space, we trained an Elastic Net model on (i) each of the three transdiagnostic dimensions and (ii) the residuals of each transdiagnostic dimension after controlling for the other two dimensions. That is, the anxious-depression residual is derived from a linear model as follows: anxious-depression ~ compulsivity + social withdrawal. We then used the selection frequencies in the same manner as above; to identify text features that were predictive of the residual and if so, whether or not that text feature was specific to that dimension, after removing the shared variance with the other dimensions.

### The effect of number of words per user on predictive performance

As a control analysis, we tested the effect of the minimum number of words per user on predictive performance. For our main analyses we chose a relatively low word count threshold per user to maximise our sample size, including participants with at least 5 days of Tweets^9^. However, there is evidence recommending that a minimum of 200 words per user be used in order to achieve stable predictive performance^74^ and several other studies have used a minimum of 500 words per user^8,91^. We thus tried three additional minimum word count per user threshold for inclusion: 200, 400, and 500 words per user. Sample size was not substantially affected by the additional inclusion criterion such that excluding participants with 200 words reduced the sample size to 945 participants, 400 words reduced the sample to 866 participants, and 500 words to 836 participants. However, to ensure that sample size differences across these minimum word thresholds did not affect our results, we down-sampled our data to the smallest sample size of 836 participants from the 500-word threshold and carried out all analyses on these subjects.

### Statistical power

Finally, we tested if we were sufficiently powered to find an effect size greater than the reported value. To interrogate this possibility, we simulated three types of datasets with 99 input features and 1 continuous target outcome with a sample size of either 1000 or 3000. We set the correlation between either 1, 10, or 20 features with the target variable at *r* = 0.32, while the other input variables had no association with the target. We chose to set *r* = 0.32, because that is approximately twice the observed effect size obtained from our depression model. Furthermore, for the datasets with greater than 1 feature associated with the target, we simulated multicollinearity among the relevant features by setting the correlation between those features at *r* = 0.50. We then ran each dataset through our Elastic Net analysis pipeline, tenfold nested cross-validation with 100 experimental runs and reported both *R*^2^ and the mean absolute error as measure of model fit. To test for the likelihood that the true effect size is larger than we report (i.e., that it is in fact *r* = 0.32) and we are missing it, we plot the proportion of cases in the 1000-person sample that performed worse than our reported predictive performance (Supplementary Fig. 7).

### Reporting summary

Further information on research design is available in the Nature Research Reporting Summary linked to this article.

## Supplementary information

Supplementary Information

Reporting Summary Checklist

## Data Availability

Processed and anonymized datasets will be made available from the corresponding author upon reasonable request from other researchers, but raw tweets cannot be made available due to the potential for re-identifying research participants.

## Code availability

The code used to analyse the data in the current study is available at: https://github.com/seanwkelley/TwitterLanguageSpecificity.
