# Supplementary material for: Machine learning of language use on Twitter reveals weak and non-specific predictions
Source: NPJ Digit Med. 2022 Mar 25;5:35. doi: 10.1038/s41746-022-00576-y (PMC8956571; doi:10.1038/s41746-022-00576-y)
Supplement: Supplementary file 1 — Supplementary Information [file 41746_2022_576_MOESM1_ESM.pdf]

Supplementary Material

Effect of minimum word count per user on the depression trained model's predictive performance. 2

Association between 9 self-report questionnaires with age and gender. 3

Bivariate correlations among 9 psychiatric questionnaires and age. 3

Predictive performance of an anxious depression trained model tested on three transdiagnostic dimensions 4

No association between depression residuals from text feature only trained model and Twitter use 5

Predictive performance of a depression model trained on subsets of Tweets 6

Histograms of 9 psychiatric scales with means and standard deviations. 6

Power to detect an effect size double the observed depression predictive performance (i.e., r = 0.32) in simulated data 7

Minimum word count per user	Sample Size	Depression R^2	Depression r
**5 days of Tweets/43 words**	836	0.010	0.13
**200**	836	-0.001	0.07
**400**	836	0.044	0.22
**500**	836	0.034	0.21

Supplementary Table 1: Effect of minimum word count per user on the depression trained model's predictive performance, controlling for sample size. The minimum word count inclusion criterion used throughout the main text is '5 days of Tweets/43 words'.

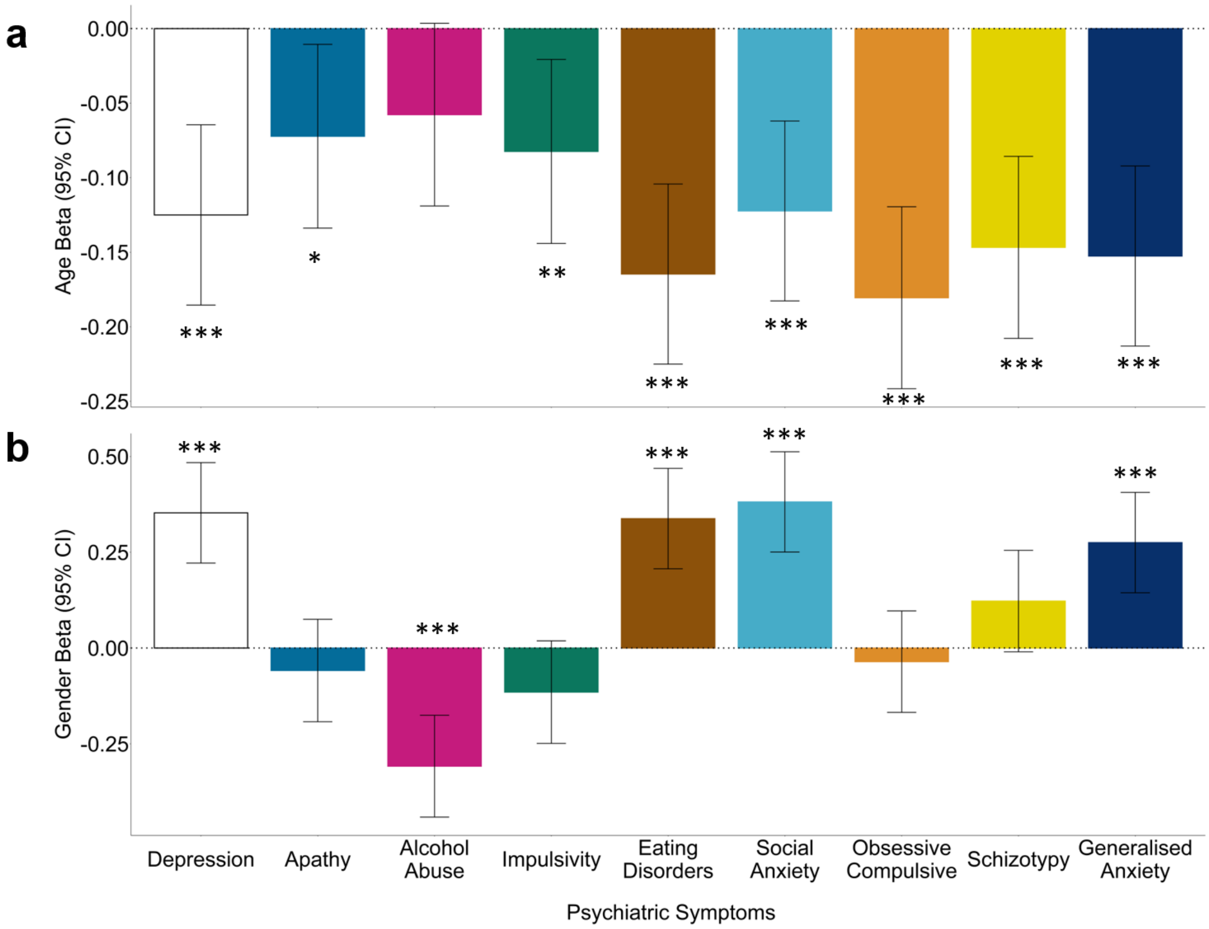

Supplementary Figure 1: Association between 9 self-report questionnaires with age and gender

a) Associations between 9 psychiatric questionnaires and age. There were significant negative associations between all questionnaires and age except for

alcohol abuse. b) Associations between questionnaires and gender. Female participants had significantly elevated levels of eating disorders, social anxiety, depression, and state anxiety compared to males. While male participants had significantly higher levels of alcohol abuse. Bars indicate a 95% confidence interval around the mean.

$*p < 0.05$, $**p<0.01$, $***p<0.001$

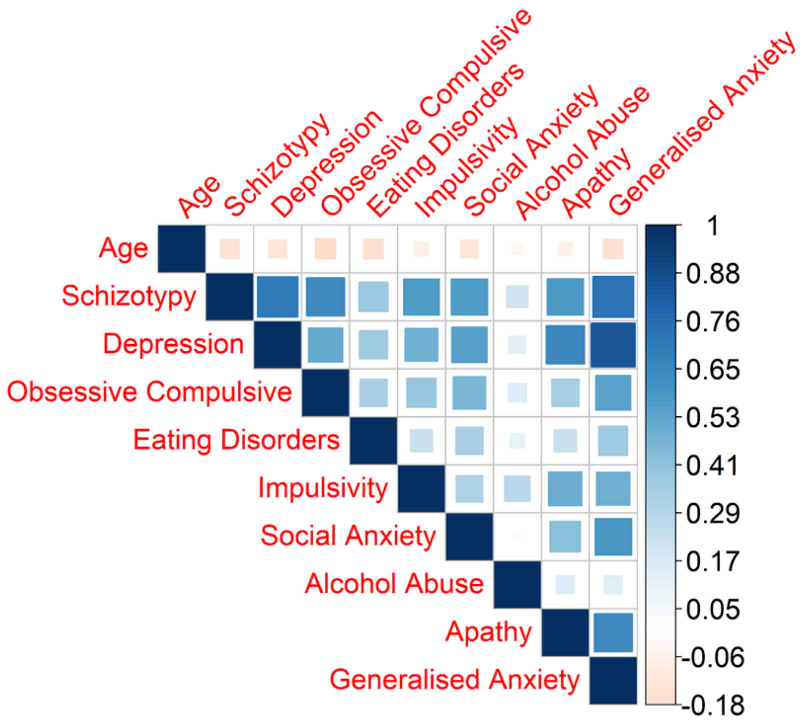

Supplementary Figure 2: Bivariate correlations among 9 psychiatric questionnaires and age

All psychiatric disorders are positively correlated with each other. Age is negatively associated with every disorder.

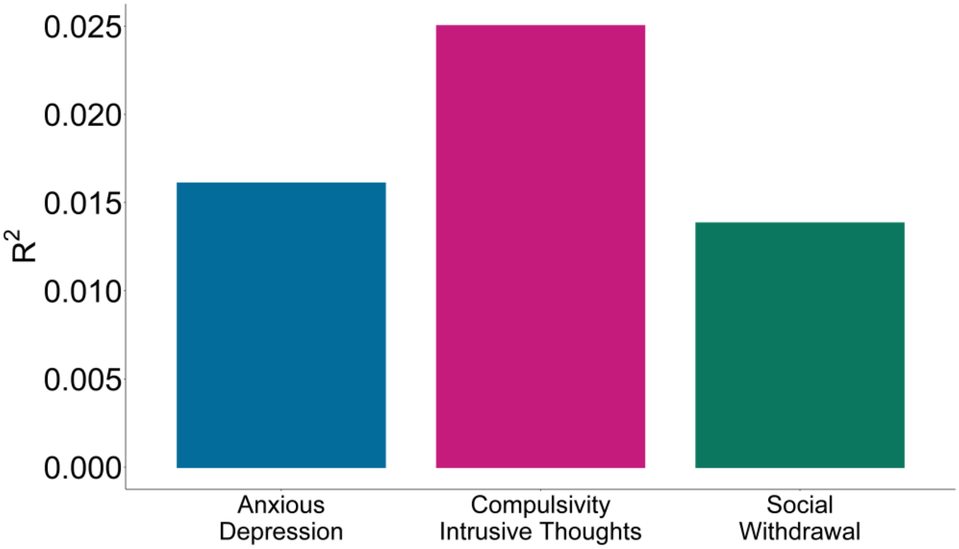

Supplementary Figure 3: Predictive performance of an anxious depression trained model tested on three transdiagnostic dimensions: anxious depression, compulsivity and intrusive thoughts, and social withdrawal

The anxious depression model performed best when tested on compulsivity and intrusive thoughts (R^2 = 0.025), but had above zero performance on all three dimensions.

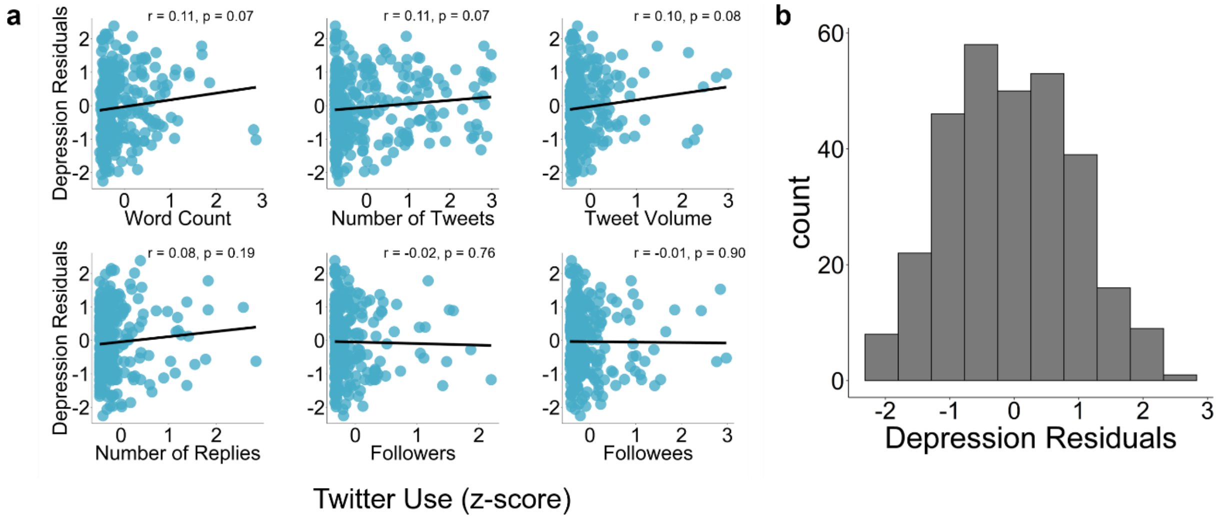

Supplementary Figure 4: No association between z-scored Twitter use and depression residuals derived from LIWC text feature model

a) Regression plots for depression residuals and z-scored Twitter use including: mean word count, total number of tweets, tweet volume, total number of replies, followers, and followees. There was no significant association between Twitter use and depression residuals (all |β| > 0.02, p > 0.05). b) Histogram of depression residuals were centered on zero (Mean = -0.05, t = -0.84 (df = 301), p = 0.40).

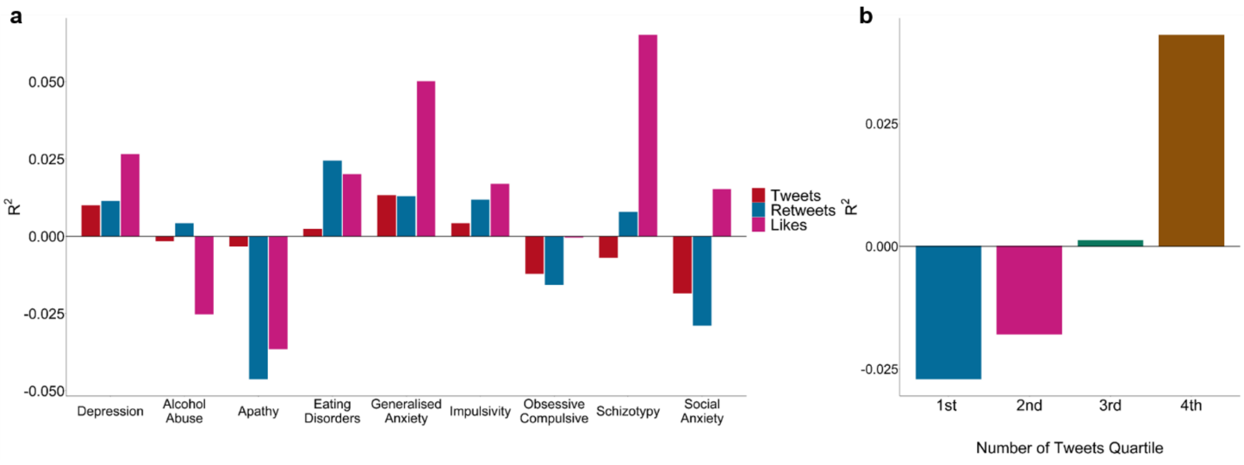

Supplementary Figure 5: Predictive performance of a depression model trained on subsets of Tweets

A) Predictive performance (R^2) of a depression model trained using only Tweets (n = 756), Retweets (n = 637), or Likes (n = 902). B) Depression model performance on four quartiles of text feature data: 1st quartile (mean Tweets = 23.5), 2nd quartile (mean Tweets = 159.2), 3rd quartile (mean Tweets = 867.2), and 4th quartile (mean Tweets = 3,625.8). Text features were divided into quartiles based on the total number of Tweets, Retweets, and Likes. Models trained on data from the 3rd and 4th quartiles had above zero performance compared to those trained on data from the lower two quartiles.

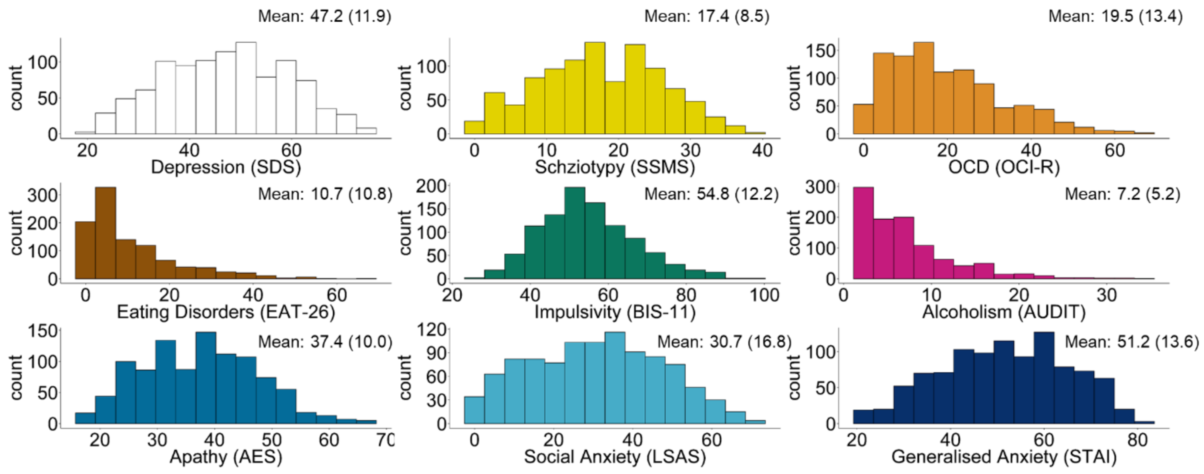

Supplementary Figure 6: Histograms of 9 psychiatric scales with means and standard deviations

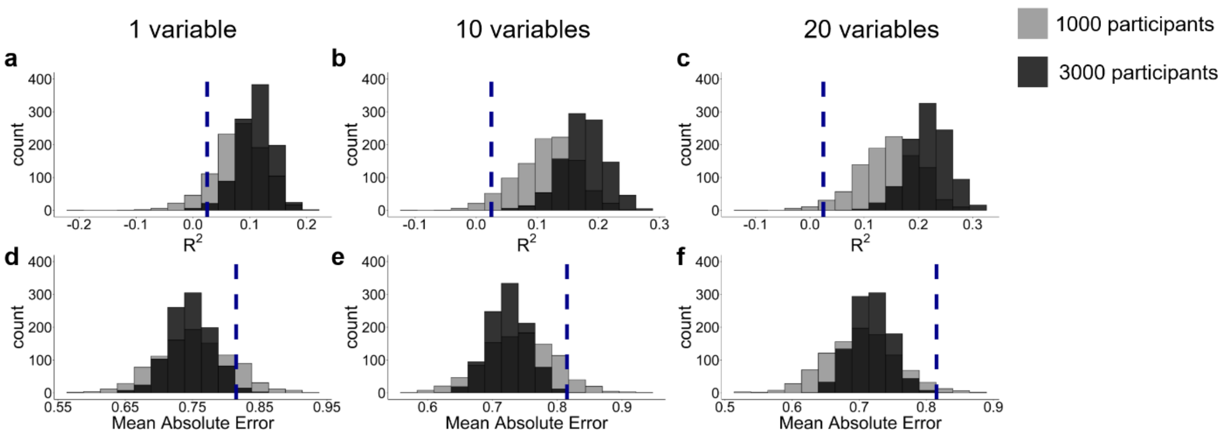

Supplementary Figure 7: Power to detect an effect size double the observed depression predictive performance (i.e., r = 0.32) in simulated data

Simulation data of 3 types of datasets with 99 input features and 1 continuous target outcome with a sample size of either 1,000 or 3,000. The correlation between either 1, 10, or 20 features with the target outcome was set to r = 0.32. In datasets with more than 1 feature, multicollinearity was simulated among the relevant features by setting the correlation between those features to r = 0.50.

When only 1 variable was associated with the target outcome, 10.6% of simulated values had a worse performance than our observed value (blue dashed line; R^2 = 0.025, MAE = 0.815) (Figure S4A and S4D). However, this percentage dropped to only 2.3% when there were 20 variables associated with the target outcome (Figure S4C and S4F). Consequently, even in the worst-case scenario, where only 1 variable is truly associated with the target outcome, we would expect to report a larger R^2 value than what we observed in our study in approximately 90% of cases. Although increasing the sample size to 3,000 participants would further reduce the likelihood to 1%, it is already

very unlikely to miss an effect size of this magnitude with a sample of 1,000. In our dataset, we have observed 51 variables significantly associated with depression severity. Thus, the likelihood of missing an effect size as large as $r = 0.32$ in our dataset is much smaller than 2.3%.
