# Supplementary material for: Machine learning of language use on Twitter reveals weak and non-specific predictions
Source: NPJ Digit Med. 2022 Mar 25;5:35. doi: 10.1038/s41746-022-00576-y (PMC8956571; doi:10.1038/s41746-022-00576-y)
Supplement: Supplementary file 2 — Reporting Summary Checklist [file 41746_2022_576_MOESM2_ESM.pdf]

nature portfolio

Corresponding author(s): Sean Kelley

Last updated by author(s): Jan 14, 2022

Reporting Summary

Nature Portfolio wishes to improve the reproducibility of the work that we publish. This form provides structure for consistency and transparency in reporting. For further information on Nature Portfolio policies, see our Editorial Policies and the Editorial Policy Checklist.

Statistics

For all statistical analyses, confirm that the following items are present in the figure legend, table legend, main text, or Methods section.

n/a	Confirmed	
☐	☒	The exact sample size (*n*) for each experimental group/condition, given as a discrete number and unit of measurement
☐	☒	A statement on whether measurements were taken from distinct samples or whether the same sample was measured repeatedly
☐	☒	The statistical test(s) used AND whether they are one- or two-sided<br>*Only common tests should be described solely by name; describe more complex techniques in the Methods section.*
☐	☒	A description of all covariates tested
☐	☒	A description of any assumptions or corrections, such as tests of normality and adjustment for multiple comparisons
☐	☒	A full description of the statistical parameters including central tendency (e.g. means) or other basic estimates (e.g. regression coefficient) AND variation (e.g. standard deviation) or associated estimates of uncertainty (e.g. confidence intervals)
☐	☒	For null hypothesis testing, the test statistic (e.g. *F*, *t*, *r*) with confidence intervals, effect sizes, degrees of freedom and *P* value noted<br>*Give P values as exact values whenever suitable.*
☒	☐	For Bayesian analysis, information on the choice of priors and Markov chain Monte Carlo settings
☒	☐	For hierarchical and complex designs, identification of the appropriate level for tests and full reporting of outcomes
☐	☒	Estimates of effect sizes (e.g. Cohen's *d*, Pearson's *r*), indicating how they were calculated

Our web collection on statistics for biologists contains articles on many of the points above.

Software and code

Policy information about availability of computer code

Data collection	We used a custom Python (3.7) script to download the last 3,200 Tweets and Likes from a participant's Twitter account using the Twitter API.
Data analysis	We used Python (3.7) to process and clean Twitter data as well as to perform ElasticNet regularization models from the scikit-learn package. We used R (4.0.0) to process the mental health questionnaire data and to prepare the data for input into the machine learning models. Finally, we used the Linguistic Inquiry and Word Count (LIWC) library which is a dictionary comprised of approximately 6,400 words and word-stems with 90 different output variables including: linguistic characteristics (e.g., articles and pronouns), psychological constructs (e.g., sadness and positive emotions), and general text information (e.g., punctuation and word count). The code used to analyse the data in the current study is available at: https://github.com/seanwkelley/TwitterLanguageSpecificity

For manuscripts utilizing custom algorithms or software that are central to the research but not yet described in published literature, software must be made available to editors and reviewers. We strongly encourage code deposition in a community repository (e.g. GitHub). See the Nature Portfolio guidelines for submitting code & software for further information.

Data

Policy information about availability of data

All manuscripts must include a data availability statement. This statement should provide the following information, where applicable:

- Accession codes, unique identifiers, or web links for publicly available datasets
- A description of any restrictions on data availability
- For clinical datasets or third party data, please ensure that the statement adheres to our policy

Processed and anonymized datasets will be made available from the corresponding author upon reasonable request from other researchers, but raw tweets cannot be made available due to the potential for re-identifying research participants.

Field-specific reporting

Please select the one below that is the best fit for your research. If you are not sure, read the appropriate sections before making your selection.

☐ Life sciences ☒ Behavioural & social sciences ☐ Ecological, evolutionary & environmental sciences

For a reference copy of the document with all sections, see nature.com/documents/nr-reporting-summary-flat.pdf

Behavioural & social sciences study design

All studies must disclose on these points even when the disclosure is negative.

Study description	This was a cross-sectional study that collected quantitative data including frequency of language use and mental health questionnaire summed scores.
Research sample	The research sample was users from ClickWorker and voluntary users interested in mental health from Twitter. Participants were included from ClickWorker to ensure that an adequate sample size was attained. Participants had a mean age of 30.5 years (SD: 10.1, range: 18-68), a majority were female (66.4%), currently employed (63.8%), and resided in either the U.K. (41%) or U.S. (46.9%). Participants tweeted an average of 21,126 words (SD: 30,204), median of 6,432 words, and a range of 43 – 163,700. In total, there were 21,252,845 words posted across the 1,006 participants. The sample is representative of Twitter users on Clickworker.
Sampling strategy	The majority of participants were randomly sampled from ClickWorker, an online worker platform, along with a small proportion of participants who voluntarily participated through Twitter. We collected data from approximately 1,000 participants in order to have 80% power to detect an effect size of Pearson's r = 0.09.
Data collection	We collected quantitative data related to language usage from Tweets, Retweets and Likes from each participant's Twitter account, Twitter metadata (e.g., number of followers), and 9 self-report mental health questionnaires. Participants were asked to provide their age, gender, country of residence, current employment status, and highest educational attainment. Participants then completed 9 different psychiatric questionnaires including the Zung depression scale (SDS), Short Scales for Measuring Schziotypy (SSMS), Obsessive Compulsive Inventory Revised (OCI-R), Eating Attitudes Test (EAT-26), Barratt Impulsiveness Scale (BIS-11), Alcohol Use Disorders Inventory Test (AUDIT), Apathy Evaluation Scale (AES), Liebowitz Social Anxiety Scale (LSAS), State-Trait Anxiety Inventory (STAI). All data was collected entirely online.
Timing	Data collection began in March 2019 and ended in May 2020
Data exclusions	99 participants were excluded due to failing an attention check and a further 345 participants were excluded for either not having at least 5 days of tweets or fewer than 50% of their tweets were in English. We chose to include participants with at least 5 days of Tweets based on the exclusion criteria implemented in Reece et al., (2017). We included participants with at least 50% of Tweets in English because the LIWC is an English based library for language analysis.
Non-participation	No participants dropped out or declined participation.
Randomization	Participants were not allocated into random experimental groups

Reporting for specific materials, systems and methods

We require information from authors about some types of materials, experimental systems and methods used in many studies. Here, indicate whether each material, system or method listed is relevant to your study. If you are not sure if a list item applies to your research, read the appropriate section before selecting a response.

Materials & experimental systems

n/a	Involved in the study	
☒	☐	Antibodies
☒	☐	Eukaryotic cell lines
☒	☐	Palaeontology and archaeology
☒	☐	Animals and other organisms
☐	☒	Human research participants
☒	☐	Clinical data
☒	☐	Dual use research of concern

Methods

n/a	Involved in the study	
☒	☐	ChIP-seq
☒	☐	Flow cytometry
☒	☐	MRI-based neuroimaging

Human research participants

Policy information about studies involving human research participants

Population characteristics	See above

Recruitment	We recruited 1,450 participants for this study. The majority of participants were recruited on Clickworker (N = 1,395), an online worker platform, and were paid €2.5 for their participation. A smaller number participated voluntarily (i.e., without payment) and were recruited through general advertising on Twitter and in print media (N = 55).
Ethics oversight	Approved was granted by the Trinity College Dublin Department of Psychology Research Ethics Committee (Approval ID: SPREC112018-32).

Note that full information on the approval of the study protocol must also be provided in the manuscript.
